# Revision of Entrophosporales, with Three Genera and an Identification Key for All Species Currently Attributed to This Order

**DOI:** 10.3390/jof11020097

**Published:** 2025-01-26

**Authors:** Gladstone Alves da Silva, Ewald Sieverding, Daniele Magna Azevedo de Assis, Bruno Tomio Goto, Mike Anderson Corazon-Guivin, Fritz Oehl

**Affiliations:** 1 Departamento de Micologia, CB, Universidade Federal de Pernambuco, Cidade Universitária, Av. da Engenharia s/n, Recife 50740-600, PE, Brazil; gladstonesilva@yahoo.com (G.A.d.S.); dma.assis@gmail.com (D.M.A.d.A.); 2 Institute of Agricultural Sciences in the Tropics (Hans-Ruthenberg Institute), University of Hohenheim, Garbenstr. 13, D-70599 Stuttgart-Hohenheim, Germany; sieverdinge@aol.com; 3 Departamento de Botânica e Zoologia, Universidade Federal do Rio Grande do Norte, Campus Universitário, Natal 59078-900, RN, Brazil; bruno.goto@ufrn.br; 4 Laboratorio de Biología y Genética Molecular, Universidad Nacional de San Martín, Jr. Amorarca N 315, Morales 22201, Peru; macorazong@unsm.edu.pe; 5 Agroscope, Competence Division for Plants and Plant Products, Ecotoxicology, Müller-Thurgau-Strasse 29, CH-8820 Wädenswil, Switzerland

**Keywords:** classification, systematics, taxonomy, arbuscular mycorrhizal fungi

## Abstract

The objective of the present study was to revise the recently described order Entrophosporales of the Glomeromycetes. The single family Entrophosporaceae had been divided into three genera, *Entrophospora*, *Claroideoglomus* and *Albahypha*, due to molecular phylogenetic or morphological analyses, but recently these three genera were combined within the type genus of the family, *Entrophospora*. Our new studies now suggest once more three genera, but *Entrophospora* and *Claroideoglomus* were not separated again. In the present study, we resurrected *Albahypha* with *A. drummondii* and *A. furrazolae* comb. nov. and established *Alborhynchus* gen. nov. with *A. walkeri* comb. nov. Morphologically, all glomoid morphs of the three genera have hyaline to white subtending hyphae with one spore wall continuous with the subtending hyphal wall. However, the genera can easily be differentiated from each other and from other glomoid species of the Glomeromycetes by the combination of the characteristics of the subtending hyphae, the staining reaction of the spore wall layers in Melzer’s reagent and phylogeny. In conclusion, the three AMF genera, currently recognized in the Entrophosporales, can unequivocally be identified by molecular phylogeny or by morphological characteristics of their spores and their subtending hyphae. An identification key distinguishes all AMF species currently attributed to Entrophosporales.

## 1. Introduction

The first systematic studies on the arbuscular mycorrhizal fungi (AMF) were presented by Gerdemann and Trappe [1] and included approximately 30 AMF species, all classified in the family Endogonaceae. This family, however, belongs to the Mucoromycota, based on current phylogenetic and morphological analyses, and not to the AM forming Glomeromycota (e.g., [2,3]). Shortly after the publication of Gerdemann and Trappe [1], two new AMF species were described under the epithets *Glomus infrequens* and *Glomus etunicatum* by Hall [4] and Becker and Gerdemann [5], respectively. In the meantime, it has been known that both species have a worldwide distribution (e.g., [6,7]). Remarkably, *G. infrequens* was soon renamed *Entrophospora infrequens* by Ames and Schneider [8] based on the spore formation within the stalks of sporiferous saccules. For *G. etunicatum*, exclusively glomoid spore formation is known [5,9,10,11].

These two fungi and a few other related species described in the meantime, such as *G. claroideum* [12], *G. lamellosum* [13] and *G. luteum* [14], or even *G. fistulosum* and *G. maculosum* [15], have had an eventful taxonomic past. Firstly, *Entrophospora* was assigned to the family Acaulosporaceae [16] due to its ‘acaulosporoid’ spore formation (sensu lato). Later, it was transferred to the family Entrophosporaceae due to its unique type of spore formation, named ‘entrophosporoid’, and having two spore walls but lacking the characteristically ‘beaded’ structure of the innermost wall of typical Acaulosporaceae species [17]. Secondly, the so-called ‘glomoid’ species mentioned above (*G. etunicatum* and related species) were shown to belong phylogenetically to the *Glomus* group B clade (e.g., [18,19]) and were therefore transferred to the genus *Claroideoglomus* of the simultaneously described Claroideoglomeraceae [20]. Morphologically, these members had all one diagnostic characteristic in common, which is the formation of white to subhyaline subtending hyphae [9]. Oehl et al. [10] went a step further, separating the *Glomus* species of the group B clade into two clades, the respective genera *Claroideoglomus* and *Albahypha*, based on molecular phylogeny and morphological differences residing at the spore base and subtending hyphae.

Surprisingly, it became increasingly obvious that the entrophosporoid *E. infrequens* and the glomoid *C. etunicatum* and *C. claroideum* did not clearly separate at the genus level, despite their different type of spore formation [10]. Based on Schüßler and Walker [20] and Oehl et al. [9], new glomoid species emerged within the old *Glomus* clade group B, such as *C. hanlinii* [21]. Other species, such as *E. baltica* [22] and *E. nevadensis* [23], had to be transferred to other AMF genera [10], since they did not belong to the old *Glomus* clade group B sensu Schüßler and Walker [20] and Oehl et al. [9]. Thereafter, it had become increasingly clear through phylogenetic studies that the families Entrophosporaceae and Claroideoglomeraceae represent the same fungal taxon and needed to be synonymized, with priority to the earlier described family Entrophosporaceae [17].

Recently, Błaszkowski et al. [11] synonymized *Claroideoglomus* with *Entrophospora* and transferred *Entrophospora* to a new order, Entrophosporales, with a single family, Entrophosporaceae. However, our findings of phylogenetic and morphological inconsistencies among members of *Entrophospora* suggests that some of them should be placed in at least two other genera of Entrophosporaceae, including *Albahypha*, described by Oehl et al. [10].

The objective of the present study was to re-analyze the phylogenetic and morphological data for all members of Entrophosporaceae and to consider the possibility of restoring the genus *Albahypha*, with *A. drummondii* and *A. furrazolae* comb. nov. and placing *E. walkeri* in a new genus based on a methodology proposed by Silva et al. [24] for the order Glomerales. The final objectives were to revise the systematics and classification of this AM fungal family and to present a novel morphological identification key for all AMF species within the family Entrophosporaceae and the order Entrophosporales.

## 2. Materials and Methods

### 2.1. Phylogenetic Analyses

To reconstruct the phylogeny, an alignment (dataset), based on partial SSU, ITS region and LSU nrDNA, was generated with AM fungal sequences from Entrophosporales (Supplementary Material, Spreadsheet S1). *Paraglomus peruvianum* (Corazon-Guivin, G.A. Silva and Oehl) was included as an outgroup. Only sequences with at least the ITS region and partial LSU nrDNA were used. The dataset was aligned in Mafft v.7 [25] using the default parameters. Prior to phylogenetic analyses, the model of nucleotide substitution was estimated using Topali 2.5 [26]. Bayesian (two runs over 5 × 10^6^ generations, with a sample frequency of 500 and a burnin value of 25%) and maximum likelihood (1000 bootstrap) analyses were performed, respectively, in MrBayes 3.1.2 [27] and PhyML [28], launched from Topali 2.5, using the best model selected by the program (GTR + G). Sequences of almost all AMF species currently attributed to the Entrophosporaceae were available to us, except for *E. hexagoni*, for which no molecular data was deposited so far in the public databases.

### 2.2. Specimen Analyses

Specimens of almost all AMF species currently attributed to the Entrophosporaceae were available to us, except for *E. hexagoni*, which, according to its protologue, might not be an *Entrophospora* species, but this is difficult to judge only from the original publication by Rhatwal and Gandhe [29]. Type specimen was analyzed by us for *E. infrequens* (photographic collection of Hall and Abbott [30], OSC, private collection of J.M. Trappe), *E. etunicata* (OSC), *E. claroidea* (OSC), *E. lamellosa* (OSC), *E. lutea* (OSC, ex type INVAM), *E. drummondii* (DPP) and *E. walkeri* (DPP). *Entrophospora argentinensis*, *E. glacialis* and *E. furrazolae* were originally described by at least one co-author of the present study [11] (see also [9,10]). Additionally, a living culture of *E. candida* had been generated in 2011 by Oehl in the Swiss collection for Arbuscular Mycorrhizal Fungi (SAF), maintained under the SAF-entry SAF112.

Specimens mounted on microscopic slides prior to 1990 were mostly mounted in lactophenol, while others were fixed with polyvinyl alcohol–lactic acid–glycerol (PVLG) or in a mixture of PVLG + Melzer’s reagent, which post-1990 were the principal fixing media [31]. Newly mounted spores from collections or cultures were fixed using the latter two fixing media or occasionally also a mixture of 1:1 lactic acid to water, in Melzer’s reagent and in water [32]. When available, spores freshly isolated from soils or bait cultures were also mounted and analysed. All spore observations and information on spore characteristics are based on spores extracted from soil, trap cultures or single or multiple spore-derived pure cultures. No information is provided from in vitro-cultured materials. Spore wall terminology follows the nomenclature of Walker [33] and Błaszkowski [34]. Analyses of the spore walls, germination structures and mycorrhizal structures were performed using compound microscopes at 100–1000 × magnifications (Zeiss Axioplan, Oberkochen, Germany; Leika DFC 295; Wetzlar, Germany). For this paper, all original species descriptions and available species emendations were considered.

## 3. Results

### 3.1. Molecular Phylogeny

**Figure 1:**
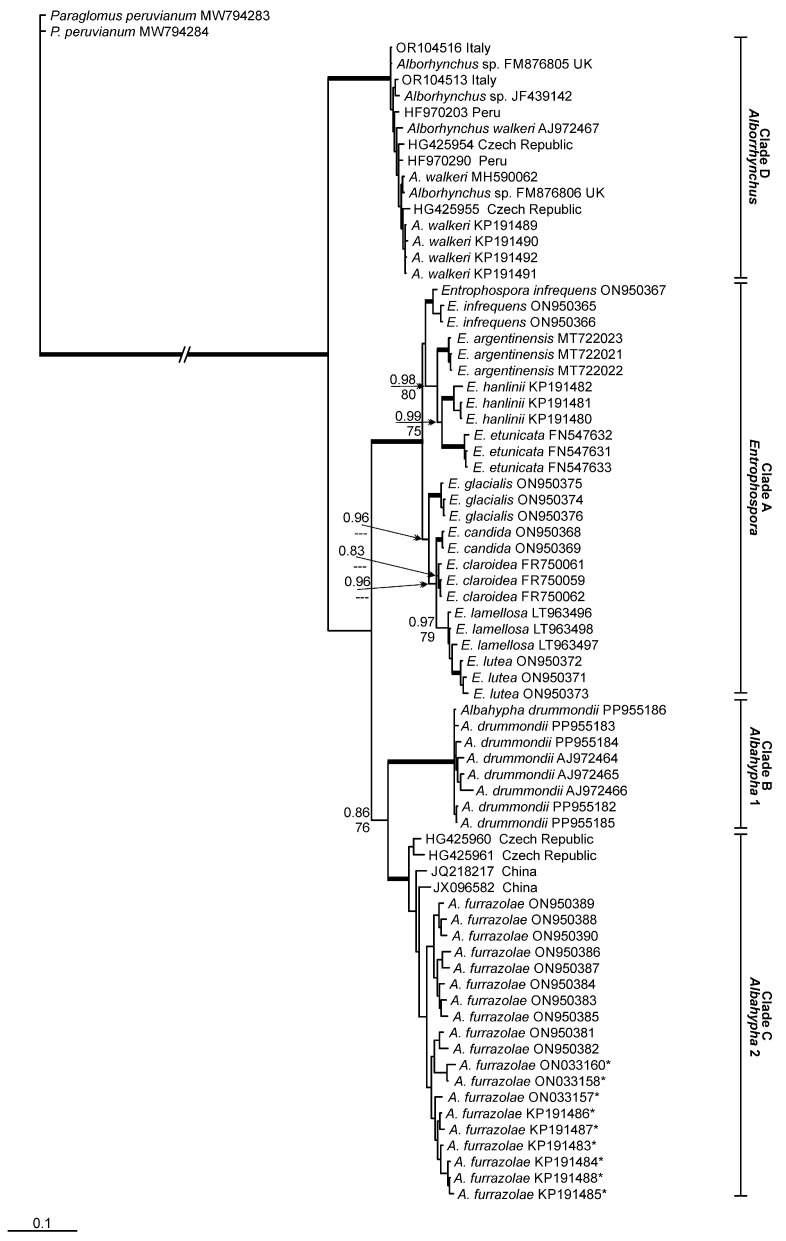
Phylogenetic tree obtained by analysis from partial SSU, ITS region and partial LSU nrDNA sequences of Entrophosporales. Sequences are labeled with their database accession numbers. Support values (from top) are from Bayesian inference (BI) and maximum likelihood (ML). Only support values of at least 70% are shown. Thick branches represent clades with more than 90% support in all analyses. The tree was rooted by *Paraglomus peruvianum*. Sequences initially assigned as *Albahypha drummondii* are indicated by *.

According to the phylogenetic analyses, Entrophosporales is divided into four different clades (Figure 1) with strong support (>90%) in maximum likelihood (ML) and Bayesian inference (BI) analyses. The first clade (‘A’) is composed by sequences from nine *Entrophospora* taxa; clade ‘B’ represents *Albahypha drummondii*. *Albahypha furrazolae* (=*E. furrazolae*) and some environmental sequences related to this species are placed in clade ‘C’. Clade ‘D’ is represented by *Alborhynchus,* a new genus represented by *A. walkeri* and environmental sequences closely related to this taxon.

The BLASTn analyses, considering the entire fragment (partial SSU, ITS region and partial LSU nrDNA), reported a maximum identity (MI) of clade ‘C’ (*A. furrazolae*) to sequences from *A. drummondii*, of 92.3% MI, and from *E. infrequens*, of 91.7% MI. The closest sequences to clade ‘B’ (*A. drummondii*), except sequences from *A. furrazolae*, belong to *E. infrequens* (90.8% MI). The nearest sequences for clade ‘D’ (*A. walkeri*) belong to *A. furrazolae* (89.4% MI).

We found five environmental sequences related to *A. furrazolae* with >95% MI. These sequences were obtained from grassland soil (JQ218217, JX096582) in China [35,36] and from roots of *Ligustrum vulgare* L. (HG425960, HG425961, HG425962) in the Czech Republic [37]. No environmental sequences related to *A. drummondii* were found. In relation to *Alborhynchus walkeri*, we found 23 environmental sequences with >95% MI. Seventeen of these sequences were obtained from rhizosphere soil of *Solanum tuberosum* L. (HF970195, HF970199, HF970200, HF970202, HF970203, HF970209, HF970210, HF970221, HF970253, HF970254, HF970288, HF970289, HF970290, HF970328, HF970329, HF970330, HF970335) in Peru [38], four from roots of *Solanum lycopersicum* L. (OR104513, OR104514, OR104515, OR104516) in Italy [39] and two from roots of *Zea mays* L. (HG425954, HG425955) in the Czech Republic [37]. Four sequences related to the new genus are from an isolate assigned as *Glomus* sp. (FM876804, FM876805, FM876806, FM876807) found in the UK [40]. Another sequence (JF439142) from an AMF isolate (assigned as *Glomus* sp.) is related to *A. walkeri*, but without information about location where it was found.

### 3.2. Morphological Analyses

The morphological analyses on species of the Entrophosporaceae have been intensified and have greatly progressed within the last 15 years, since Oehl et al. [9,10] not only focused on the spores but also considered the morphology of subtending hyphae (SH) of AMF species forming glomoid spores. Outstandingly, Błaszkowski et al. [11] revealed that *E. infrequens* does not only form spores within the neck of sporiferous saccules but also directly on subtending hyphae. They furthermore revealed that the morphology of the glomoid spores of *E. infrequens* fits well with the morphology of all other species, for which exclusive glomoid spore formation has been observed in the *Entrophospora* clade. The diagnostic feature of glomoid morphs in Entrophoporaceae is the formation of hyaline to subhyaline or whitish SH, whose SH wall layers are continuous with the spore wall layers inclusively of the structural wall layer.

With respect to the major clades of the Entrophosporaceae (Figure 1), the findings of Oehl et al. [10] are repeated and elaborated here. Glomoid species of the *Entrophospora* clade regularly have hyaline to subhyaline or whitish funnel-shaped SH, and generally, the colour-change towards the pigmented spore wall layers is exactly at the spore base, if the species form pigmented spores. In this clade, the structural wall layer (often L2 or L3) is >2.5 times thicker at the spore base than 10–25 μm of the transition between SH and mycelia hypha [9,10]. Spores of the *Albahypha* clade have cylindrical to sometimes slightly funnel-shaped SH, with structural wall layers (generally swl2) that are <2.0 times thicker at spore base than 10–25 μm distant [9,10]. Finally, spores of *A. walkeri*, hereafter transferred to the new genus *Alborhynchus*, have slightly funnel-shaped to rarely cylindrical hyphae, whose structural wall layer usually are about 2.0–2.5 times thicker at spore base than 10–25 μm distant at the transition between SH and mycelia hypha. Another obvious morphological difference, so far detected between species of *Albahypha* and *Alborhynchus*, is the lack of staining reaction on the innermost, flexible layer in *A. walkeri*, when compared to the greyish rose staining reaction in both *A. drummondii* and *A. furrazolae* (see and compare in Błaszkowski et al. [11,41]).

### 3.3. Taxonomy

Phylogenetically and morphologically, three distinct major clades were found. Hereafter, *Albahypha* [10] is resurrected, and a new, third genus (*Alborhynchus*) is described in the Entrophosporaceae. *Entrophospora furrazolae* is transferred to *Albahypha*, while *A. walkeri* is transferred to *Alborhynchus* and becomes the type species of this new genus.

**Entrophosporales** Błaszk., Sánchez-García, B.T. Goto & Magurno, Frontiers in Microbiology 13 (no. 962856): 10 (2022)

Description: see Błaszkowski et al. [11]

Type family: **Entrophosporaceae** Oehl & Sieverd., J. Appl. Bot. 80: 73 (2006)

Description: see Błaszkowski et al. [11]. SH generally pronounced funnel-shaped, as SH >1.5 times wider at the spore base than towards the mycelia hyphae; the structural wall layer is >2.5 times thicker at spore base than at the transition between SH and mycelia hypha.

Type genus: ***Entrophospora*** R.N. Ames & R.W. Schneid., Mycotaxon 8 (2): 347 (1979)

Description: see Błaszkowski et al. [11]

Type species: *Entrophospora infrequens* (I.R. Hall) R.N. Ames & R.W. Schneid., Mycotaxon 8 (2): 348 (1979)

Basionym: *Glomus infrequens* I.R. Hall, Transactions of the British Mycological Society 68 (3): 345 (1977)

Other genera: *Albahypha* Oehl et al., *Alborhynchus* Oehl et al.

***Albahypha*** Oehl, G.A. Silva, B.T. Goto & Sieverd., Mycotaxon 117: 308 (2011)

Emended description: Spores formed generally singly in soil or rarely in roots; SH white, rarely subhyaline, generally 1.0–1.2 times wider at spore base than 10–25 μm below towards the mycelia hyphae, giving a cylindrical or slightly flared appearance, with structural wall layers that are <2.0 times thicker at the spore base than 10–25 μm below. Spores with one wall of 1–4 layers; pore closure at spore base or in SH often with a septum that may arise from the structural layer, an adherent innermost, (semi-)flexible layer or both innermost layers. Innermost layers beneath the structural layer stain pinkish to reddish grey to purple in Melzer’s reagent.

Type species: *Albahypha drummondii* (Blaszk. & Renker) Sieverd., Oehl, B.T. Goto and G.A. Silva, Mycotaxon 117: 308 (2012)

Basionym: *Glomus drummondii* Błaszk. & Renker, Mycological Research 110 (5): 559 (2006)

Synonym: *Claroideoglomus drummondii* (Błaszk. & Renker) C. Walker & A. Schüßler, The Glomeromycota, a species list with new families and new genera (Gloucester): 22 (2010)

Synonym: *Entrophospora drummondii* (Błaszk. & Renker) Błaszk., Niezgoda, B.T. Goto & Magurno, Frontiers in Microbiology 13 (no. 962856): 13 (2022)

*Albahypha furrazolae* (Magurno, Niezgoda, B.T. Goto & Błaszk.) G.A. Silva, B.T. Goto, Corazon-Guivin, Sieverd. & Oehl, comb. nov.

MycoBank MB 856947

Basionym: *Entrophospora furrazolae* Magurno, Niezgoda, B.T. Goto & Błaszk., Frontiers in Microbiology 13 (no. 962856): 17 (2022)

***Alborhynchus*** gen. nov. Oehl, B.T. Goto, Corazon-Guivin, Sieverd. & G.A. Silva

MycoBank MB 856948

Description: Spores formed generally singly in soil or rarely in roots; SH white, rarely subhyaline, generally 1.2–1.5 times wider at spore base than 10–25 μm below towards the transition between SH and mycelia hyphae, giving a slightly funnel-shaped or cylindrical appearance; structural spore wall layers usually about 2.0–2.5 times thicker at spore base than 10–25 μm below. Spores with one wall of 1–4 layers; pore closure at spore base or in SH, often with a septum that may arise from the structural layer, an adherent innermost, (semi-)flexible layer or both innermost layers. Innermost layers beneath the structural layer do not stain in Melzer’s reagent.

Etymology: *albus* = white, *rhynchus* = referring to the glomoid spore formation on subtending hyphae, which resemble beaks of insectivore birds, when compared to SH of *E. etunicata* and related species having SH rather resembling larger beaks of granivore birds.

Type species: *Alborhynchus walkeri* (Błaszk. & Renker) G.A. Silva, B.T. Goto, Corazon-Guivin, Sieverd. & Oehl, comb. nov.

MycoBank MB 856949

Basionym: *Glomus walkeri* Błaszk. & Renker, Mycological Research 110 (5): 563 (2006)

Synonym: *Claroideoglomus walkeri* (Błaszk. & Renker) C. Walker & A. Schüßler, The Glomeromycota, a species list with new families and new genera (Gloucester): 22 (2010)

Synonym: *Albahypha walkeri* (Błaszk. & Renker) Sieverd., Oehl, B.T. Goto & G.A. Silva, Mycotaxon 117: 309 (2012)

Synonym: *Entrophospora walkeri* (Błaszk. & Renker) Błaszk., Niezgoda, B.T. Goto & Magurno, Frontiers in Microbiology 13 (no. 962856): 13 (2022)

### 3.4. Morphological Identification Key to the Entrophosporales Species

Here, the first morphological identification key is presented for the species attributed to the order Entrophosporales. We are currently unsure if *E. hexagoni* [29] belongs to this or another order within the Kingdom Fungi, due to the lack of molecular analyses and the unavailability of type specimens or mycorrhizal structures. However, as this species, based on its description, does not interfere with any other AMF species, *E. hexagoni* was included here in this key.

1 Spores formed within the stalk of a sporiferous saccule = entrophosporoid …..….. 2

1′ Spores formed on hyaline subtending hyphae, wall layers of SH, including the structural wall layer are continuous with spore wall layers (=glomoid) ….……………… 3

2 Spores with hyaline hexagonal reticulated net ornamentation on the sporiferous saccule: ……………………………………….... *Entrophospora hexagoni* Rhatwal and Gandhe

2′ Spores ornamented with rounded projections growing on the golden yellow to orange-brown to brown wall layer; at the top, the projections often have a convex central depression; spores 95–175 μm: ………. entrophosporoid morph of *Entrophospora infrequens* (I.R. Hall) R.N. Ames and R. W. Schneid. emend. Błaszk. et al.

3 Subtending hyphae generally pronounced funnel-shaped (SH > 1.5 times wider at the spore base than towards the mycelia hyphae, although pore channel rather small up to the spore base (=glomoid morph of *Entrophospora*) ………..……….……………………. 4

3′ Subtending hyphae not pronounced funnel-shaped (SH about 1.0–1.5 times wider at the spore base than towards the mycelia hyphae) ……..……………………………….. 12

4 Spores with 2 spore wall layers ……………..…………………………………………. 5

4′ Spores with >2 spore wall layers ………..……………………….……….…………… 8

5 Spores white to pale yellow to olive yellow …………………….……………………. 6

5 Spores brown to reddish-brown or rarely yellow-brown, 68–160 μm, L1 hyaline, evanescent; L2 yellow brown to brown to dark reddish brown: ......………………………………...… *E. etunicata* (W.N. Becker and Gerd.) Błaszk. et al.

6 Spores generally <70 μm, i.e., 41–69 × 36–64 μm; subhyaline to pastel yellow, with two permanent layers; L1 unit, semi-flexible, hyaline to yellowish white, rarely slightly deteriorated in its upper part; L2, laminate, semi-flexible, hyaline to pastel yellow, 1.2–3.4 μm thick; in Melzer’s L2 turns yellow:..……… glomoid morph of *Entrophospora infrequens*

6′ Spores generally >75 μm …...…………………..……………………………………… 7

7 Spores white to very pale yellow, 85–160 μm; L1 mucilaginous; L2 hyaline to pale yellow; L1 stains pale pink in Melzer’s, when young: ……………………...…. *E. candida* (Furrazola et al.) Błaszk. et al.

7′ Spores pale yellow to olive yellow, 70–88 × 60–80 μm; L1 mucilaginous; L2 pale yellow to olive yellow; L1 occasionally stains pinkish white to orange-red in Melzer’s: ………………………...……………….……...…………. *E. hanlinii* (Błaszk. et al.) Błaszk. et al.

8 Spores hyaline to white yellow or white-creamy to ochre ….………………………. 9

8′ Spores yellow to yellow brown ……………………………….…………..………….. 10

9 Spores becoming creamy to yellow-brown when aging, 72–145 × 59–126 μm, with four layers; L1 mucilagenous; L2 hyaline; L3 laminate whitish to pale yellow; L4 hyaline to pale yellow; L1 staining pink to reddish purple in Melzer’s: …….…………… *E. claroidea* (N.C. Schenck and G.S. Sm.) Błaszk. et al.

9′ Spores hyaline to light yellow, 122–162 × 98–142 μm, with 3–4 layers; L1 evanescent; L2 hyaline, semi-persistent to persistent, with a foliated, lamellate outer surface; L3 laminate, light yellow to lemon; L4 hyaline, flexible: …………….……..… *E. lamellosa* (Dalpé et al.) Błaszk. et al.

10 Spores with 3–4 layers, generally 1–2 permanent layers ……………...…………... 11

10′ Spores with five permanent layers, yellowish white to yellowish brown, 77–101 × 90–123 μm; L1-3 semi-flexible, hyaline to yellowish white, surface occasionally with small, local thickenings rendering the spore surface slightly wavy; L4 laminate, but semi-flexible, yellowish white to yellowish brown; L5 flexible; L1–L5 not staining in Melzer’s reagent: ……...…………………………………………………………………….. *E. glacialis* Zubek et al.

11 Spores pale yellow to dark yellow with a brownish tint, 65–170 μm, with four layers; L1 mucilagenous, also L2 evanescent; L3 yellow to dark yellow, laminate; L4 flexible; only L1 staining pinkish red in Melzer’s: ……... *E. lutea* (L.J. Kenn. et al.) Błaszk. et al.

11′ Spores 85–115 × 100–140 μm, with three layers; L1, evanescent, semi-flexible, becoming roughened with age; L2 laminate, but semi-flexible, yellowish white, uneven in thickness; L3 permanent, semi-flexible, hyaline to light yellow; L1–3 do not stain in Melzer’s reagent: …...……………………………………...……... *E. argentinensis* Błaszk. et al.

12 Innermost, flexible layer staining reddish white to greyish rose or pink in Melzer’s reagent: *Albahypha* Oehl et al. ………....…………………..…………………………………... 13

12′ Innermost layer not staining in Melzer’s reagent: *Alborhynchus* Oehl et al. Spores white to pale yellow, 75–110 × 60–90 μm, L1 semi-permanent; L2 laminate, hyaline to pale yellow; L3 flexible, hyaline; L1 staining reddish white to grayish rose in Melzer’s: …………...………….………........ *Alborhynchus walkeri* (Błaszk. and Renker) G.A. Silva et al.

13 Spores hyaline to yellowish white; 50–57 × 60–74 μm, with four layers; L1 mucilaginous, evanescent; L2 uniform, permanent, flexible to semi-flexible; L3 laminate, permanent, semi-flexible, hyaline to yellowish white; L4 flexible, hyaline; L1 and L4 frequently stain reddish white to greyish rose and reddish white to pastel pink in Melzer’s reagent: ……………………………….. *Albahypha furrazolae* (Magurno et al.) G.A. Silva et al.

13′ Spores pastel yellow to maize yellow, 63–98 × 50–80 μm, with three layers; L1 mucilaginous, flexible, hyaline; L2 laminate, pastel yellow; L3 flexible, hyaline; only L3 staining reddish white to greyish rose in Melzer’s: …………………… *A. drummondii* (Błaszk. and Renker) Sieverd. et al.

## 4. Discussion

Our phylogenetic analyses distinguished four well-supported clades in Entrophosporales (*Entrophospora*, *Albahypha* 1 and *Albahypha* 2, *Alborhynchus*). According to Silva et al. [24], in the order Glomerales, all genera differ molecularly by about 10% MI, considering the partial rDNA gene. Our findings indicate that the new genus in Entrophosporaceae (here described as *Alborhynchus*) demonstrates this pattern (as compared to the other genera in the family) that was already applied to separate genera in Glomerales [24]. The nearest species to *Alborhynchus* was *Albahypha furrazolae* (89.4% MI), and the next was *E. infrequens* (89.0% MI), while *Albahypha drummondii* presented 88.1% MI to *Alborhynchus*.

Our phylogenetic tree places *Alborhynchus* as a sister group to *Albahypha* and *Entrophospora*, without a close relation to either of these two clades. The new genus is also morphologically distinct from the two other genera of Entrophosporaceae. Consequently, *Alborhynchus* is supported by phylogeny (95% ML and 0.99 BI), MI divergence from other genera (about 10%) and morphological characteristics. The phylogenetic differences between the three genera can be unequivocally confirmed with the morphological differences among *Entrophospora*, *Albahypha* and *Alborhynchus*: *Entrophospora* species can be bi-morphic, and the subtending hyphae of the glomoid morph are pronouncedly funnel-shaped. The SH of *Alborhynchus* are slightly funnel-shaped to rarely cylindrical, and the SH of *Albahypha* spores are generally cylindrical (see taxonomy section). Secondly, the wall-thickening of the SH towards the spore base decreases in the three genera: *Entrophospora* > *Albahypha* > *Alborhynchus*. Furthermore, the two *Albahypha* species can be easily distinguished from *Alborhynchus* by the staining reaction of the innermost flexible wall layers in Melzer’s reagent, which is absent in *Alborhynchus*.

Błaszkowski et al. [11] synonymized *Claroideoglomus* and *Albahypha* with *Entrophospora* according to the priority of the first described (i) species, (ii) genus and (iii) family of this order. However, the tree generated by those authors showed the same four main clades, found by us, with strong support (Figure 1). The same groups/clades were also well supported by Tedersoo et al. [3]. The minimal MI divergence among *Albahypha* and *Entrophospora* was about 8.5%, but it was not possible for us to place *Albahypha* together with *Entrophospora*, considering the lack of support for this grouping in the tree. Similar findings are shown in the tree constructed by Tedersoo et al. [3]. In our opinion, each order in Glomeromycota has its own evolutionary pathway. Thus, the divergence in Entrophosporales, to determine genera in the order, can be different and lower than that observed in Glomerales.

The clades *Albahypha* 1 and 2 here share a common major clade, with low support for BI (0.86) and moderate-to-good support for ML (76%). In the tree constructed by Tedersoo et al. [3], *A. drummondii* and *A. furrazolae* are clustered together with strong support (95.7/100%). In Błaszkowski et al. [11], *A. drummondii* was not grouped with *A. furrazolae*. Considering these results, we are unsure if *A. furrazolae* represents a genus separated from *Albahypha.* For now, we decided to not describe another new genus based on this species. This decision is absolutely acceptable with respect to spore and subtending hypha morphology of these two *Albahypha* species, as both are morphologically closely related.

## Data Availability

All data (sequences) presented in this article are in GenBank (https://www.ncbi.nlm.nih.gov).

## Supplementary Materials

The following supporting information can be downloaded at: https://www.mdpi.com/article/10.3390/jof11020097/s1, Spreadsheet S1: GenBank accession numbers for the sequences used in this study.

## References

[1] Gerdemann J.W., Trappe J.M. (1974). The Endogonaceae of the Pacific Northwest. Mycol. Mem..

[2] Bidartondo M.I., Read D.J., Trappe J.M., Merckx V., Ligrone R., Duckett J.G. (2011). The dawn of symbiosis between plants and fungi. Biol. Lett..

[3] Tedersoo L., Magurno F., Alkahtani S., Mikryukov V. (2024). Phylogenetic classification of arbuscular mycorrhizal fungi: New species and higher-ranking taxa in *Glomeromycota* and Mucoromycota (class Endogonomycetes). MycoKeys.

[4] Hall I.R. (1977). Species and mycorrhizal infections of New Zealand Endogonaceae. Trans. Br. Mycol. Soc..

[5] Becker W.N., Gerdemann J.W. (1977). *Glomus etunicatus* sp. nov. Mycotaxon.

[6] Silva E.M., de Melo N.F., Mendes A.M.S., de Araujo F.P., Maia L.C., Yano-Melo A.M. (2015). Response of *Passiflora setacea* to mycorrhization and phosphate fertilization in a Semiarid Region of Brazil. J. Plant Nutr..

[7] Moebius-Clune D.J., Moebius-Clune B.N., van Es H.M., Pawlowska T.E. (2013). Arbuscular mycorrhizal fungi associated with a single agronomic plant host across the landscape: Community differentiation along a soil textural gradient. Soil Biol. Biochem..

[8] Ames R.N., Schneider R.W. (1979). *Entrophospora*, a new genus in the Endogonaceae. Mycotaxon.

[9] Oehl F., Silva G.A., Goto B.T., Sieverding E. (2011). *Glomeromycota*: Three new genera and glomoid species reorganized. Mycotaxon.

[10] Oehl F., Silva G.A., Sánchez-Castro I., Goto B.T., Maia L.C., Vieira H.E.E., Barea J.-M., Sieverding E., Palenzuela J. (2011). Revision of Glomeromycetes with entrophosporoid and glomoid spore formation with three new genera. Mycotaxon.

[11] Błaszkowski J., Sánchez-García M., Niezgoda P., Zubek S., Fernández F., Vila A., Al-Yahya’ei M.N., Symanczik S., Milczarski P., Malinowski R. (2022). A new order, Entrophosporales, and three new *Entrophospora* species in *Glomeromycota*. Front. Microbiol..

[12] Schenck N.C., Smith G.S. (1982). Additional new and unreported species of Mycorrhizal fungi (Endogonaceae) from Florida. Mycologia.

[13] Dalpé Y., Koske R.E., Tews L.L. (1992). *Glomus lamellosum* sp. nov.: A new Glomaceae associated with beach grass. Mycotaxon.

[14] Kennedy L.J., Stutz J.C., Morton J.B. (1999). *Glomus eburneum* and *G. luteum*, two new species of arbuscular mycorrhizal fungi, with emendation of *G. spurcum*. Mycologia.

[15] Walker C., Vestberg M. (1998). Synonymy amongst the arbuscular mycorrhizal fungi: *Glomus claroideum*, *G. maculosum*, *G. multisubstenum* and *G. fistulosum*. Ann. Bot..

[16] Morton J.B., Benny G.L. (1990). Revised classification of arbuscular mycorrhizal fungi (Zygomycetes): A new order, Glomales, two new suborders, Glomineae and Gigasporinae, and two families, Acaulosporaceae and Gigasporaceae, with an emendation of Glomaceae. Mycotaxon.

[17] Sieverding E., Oehl F. (2006). Revision of *Entrophospora* and description of *Kuklospora* and *Intraspora*, two new genera in the arbuscular mycorrhizal Glomeromycetes. J. Appl. Bot. Food Qual..

[18] Schüßler A., Schwarzott D., Walker C. (2001). A new fungal phylum, the *Glomeromycota*: Phylogeny and evolution. Mycol. Res..

[19] Furrazola E., Herrera-Peraza R., Kaonongbua W., Bever J.D. (2010). *Glomus candidum*, a new species of arbuscular mycorrhizal fungi from North American grassland. Mycotaxon.

[20] Schüßler A., Walker C. (2010). The Glomeromycota. A Species List with New Families and New Genera.

[21] Błaszkowski J., Chwat G., Góralska A. (2015). *Acaulospora ignota* and *Claroideoglomus hanlinii*, two new species of arbuscular mycorrhizal fungi (*Glomeromycota*) from Brazil and Cuba. Mycol. Prog..

[22] Błaszkowski J., Madej T., Tadych M. (1998). *Entrophospora baltica* sp. nov. and *Glomus fuegianum*, two species in the Glomales from Poland. Mycotaxon.

[23] Palenzuela J., Barea J.M., Ferrol N., Azcón-Aguilar C., Oehl F. (2010). *Entrophospora nevadensis*, a new arbuscular mycorrhizal fungus, from Sierra Nevada National Park (southeastern Spain). Mycologia.

[24] Silva G.A., Corazon-Guivin M.A., Assis D.M.A., Oehl F. (2023). *Blaszkowskia*, a new genus in Glomeraceae. Mycol. Prog..

[25] Katoh K., Rozewicki J., Yamada K.D. (2019). MAFFT online service: Multiple sequence alignment, interactive sequence choice and visualization. Brief. Bioinform..

[26] Milne I., Lindner D., Bayer M., Husmeier D., McGuire G., Marshall D.F., Wright F. (2009). TOPALi v2: A rich graphical interface for evolutionary analyses of multiple alignments on HPC clusters and multi-core desktops. Bioinformatics.

[27] Ronquist F., Huelsenbeck J.P. (2003). MrBayes 3: Bayesian phylogenetic inference under mixed models. Bioinformatics.

[28] Guindon S., Gascuel O. (2003). A simple, fast, and accurate algorithm to estimate large phylogenies by maximum likelihood. Syst. Biol..

[29] Rhatwal S., Gandhe R.V. (2009). *Entrophospora hexagonii*, a new arbuscular mycorrhizal fungal species from India. J. Mycol. Pl. Pathol..

[30] Hall I.R., Abbott L.K. (1979). Photographic Slide Collection Illustrating Features of the Endogonaceae.

[31] Brundrett M., Melville L., Peterson L. (1994). Practical Methods in Mycorrhizal Research.

[32] Spain J.L. (2003). Emendation of *Archaeospora* and of its type species, *Archaeospora trappei*. Mycotaxon.

[33] Walker C. (1983). Taxonomic concepts in the Endogonaceae: Spore wall characteristics in species descriptions. Mycotaxon.

[34] Błaszkowski J. (2012). Glomeromycota.

[35] Xiang D., Chen B., Li H. (2015). Specificity and selectivity of arbuscular mycorrhizal fungal polymerase chain reaction primers in soil samples by clone library analyses. Acta Agric. Scand. Sect. B Soil Plant Sci..

[36] Yang W., Zheng Y., Gao C., He X., Ding Q., Kim Y., Rui Y., Wang S., Guo L.-D. (2013). The arbuscular mycorrhizal fungal community response to warming and grazing differs between soil and roots on the Qinghai-Tibetan Plateau. PLoS ONE.

[37] Sýkorová Z., Rydlová J., Slavíková R., Ness T., Kohout P., Püschel D. (2016). Forest reclamation of fly ash deposit: A field study on appraisal of mycorrhizal inoculation. Restor. Ecol..

[38] Senés-Guerrero C., Torres-Cortés G., Pfeiffer S., Rojas M., Schüßler A. (2014). Potato-associated arbuscular mycorrhizal fungal communities in the Peruvian Andes. Mycorrhiza.

[39] Pellegrino E., Arcidiacono M., Francini A., Ercoli L. (2024). Arbuscular mycorrhizal fungi with contrasting life-history strategies differently affect health-promoting compounds in field-grown tomato by changing arbuscule occurrence and mycorrhizal assemblages in roots. Biol. Fertil. Soils.

[40] Krüger M., Stockinger H., Krüger C., Schüßler A. (2009). DNA-based species level detection of *Glomeromycota*: One PCR primer set for all arbuscular mycorrhizal fungi. New Phytol..

[41] Błaszkowski J., Renker C., Buscot F. (2006). *Glomus drummondii* and *G. walkeri*, two new species of arbuscular mycorrhizal fungi (*Glomeromycota*). Mycol. Res..

